# Ion-ing out the genetic variants of Kir2.1

**DOI:** 10.7554/eLife.80718

**Published:** 2022-07-11

**Authors:** Braden S Fallon, Justin G English

**Affiliations:** 1 Department of Biochemistry, University of Utah Salt Lake City United States

**Keywords:** deep mutational scanning, ion channel, folding, gating, high-throughput, variant effect prediction, Mouse

## Abstract

Deep mutational scanning provides new insights into how mutations alter the expression and activity of the potassium ion channel Kir2.1, which is associated with many diseases.

**Related research article** Coyote-Maestas W, Nedrud D, He Y, Schmidt D. 2022. Determinants of trafficking, conduction, and disease within a K+ channel revealed through multiparametric deep mutational scanning. *eLife* **11**:e76903. doi: 10.7554/eLife.76903.

Every time a cell in your body divides, it copies the three billion letter message stored in its DNA. This process is prone to errors and can accidentally introduce mistakes into the genetic code. To counteract this, human cells proofread the copied DNA sequence and correct any inaccuracies (Kunkel, 2004). But typos sometimes fly under the radar, leading to mutations that can alter the overall meaning of the message encoded within the cell’s DNA.

Clinicians can rapidly and easily identify these errors using high-throughput DNA sequencing techniques. However, determining how individual mutations affect the message within DNA is more challenging. This is because some mutations will not change a gene’s activity, while others may substantially impact the function of the protein it encodes, potentially resulting in disease. Traditionally, the best way to study the impact of a mutation was to perform functional studies: researchers made a single mutation, tested it in different types of laboratory experiments, and reported the effect (Jackson et al., 2018). More recently, large-scale analysis techniques that can measure the consequences of all potential mutations in a specific gene have grown in popularity. These approaches (called deep mutational scanning, or DMS for short) allow researchers to make every typo the cell could make in a gene and determine the impact of all such errors in parallel (Fowler et al., 2010).

While DMS has been applied to several genes, these studies have typically only measured a singular output, such as how mutations impact a specific cell behavior or alter the protein encoded within the gene (Jones et al., 2020; Majithia et al., 2016; Starr et al., 2020). Now, in eLife, Willow Coyote-Maestas, David Nedrud, Yungui He and Daniel Schmidt report how they used DMS to characterize how mutations in the gene coding for an ion channel called Kir2.1 impact the function of this protein and its expression simultaneously (Coyote-Maestas et al., 2022).

Kir2.1 belongs to a family of proteins called inward-rectifying potassium channels (Kirs for short) that are studded throughout the cell surface and help stabilize the membrane potential of cells. Mutations in the genes encoding the Kir channels have been linked with various diseases, including diabetes, heart dysfunction, seizures and paralysis (Zangerl-Plessl et al., 2019). However, only a small number of mutations in Kir2.1 have been clinically reported, and how they affect the behavior of the channel is poorly understood.

Coyote-Maestas et al. therefore set out to employ DMS on the Kir2.1 channel, making this the largest disease-associated complex that DMS has ever been applied to. The team (who are based at the University of Minnesota) used this approach to introduce genetic mutations that altered the sequence of amino acids within Kir2.1. Each building block was swapped with every other possible amino acid to create a library of mutants that were then individually inserted into cells grown in the laboratory. To examine how many Kir2.1 channels were expressed at the cell surface, a marker that can be fluorescently labeled was inserted into the genetic sequence of the channel. For changes in functionality, a dye was used that measures membrane potential (Figure 1).

**Figure 1.**
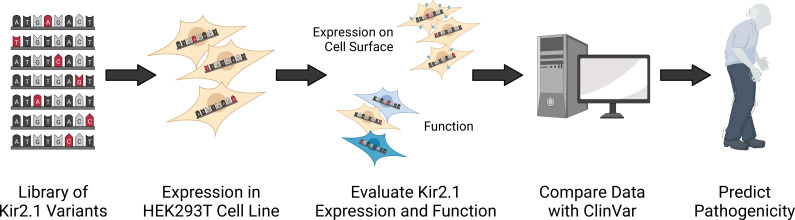
Schematic showing how deep mutational scanning was applied to Kir2.1. Mutants of the potassium channel Kir2.1 were generated by modifying its DNA sequence so that every amino acid in the protein was swapped for every other possible amino acid. These genetic variants were then integrated and expressed in human cells (called HEK293T) grown in the laboratory. Further experiments were then carried out to determine whether the mutations altered the number of channels expressed on the cell surface, and/or the function of Kir2.1. Mutants were then categorized based on how they impacted the behavior of Kir2.1 and the results were compared to a clinical dataset (called ClinVar) containing mutations found in patients. Genetic variants in the database with no assigned clinical significance were then evaluated and given a pathogenicity score indicating how likely the mutation is to cause disease based on the results from the DMS experiment.

Image credit: Braden Fallon; Figure created using BioRender.

The data revealed that mutations that decreased the expression of Kir2.1 resided in parts of the genome that are responsible for trafficking the channel to the cell surface, or for folding the protein into the right shape. Furthermore, mutations that impacted the formation of the pore in the protein (which allows potassium ions to pass through) were detrimental to the overall expression of Kir2.1. The data also identified segments of Kir2.1 that prevent faulty channels from reaching the cell surface, some of which had already been detected in previous studies (Li et al., 2016; Ma et al., 2011).

Next, Coyote-Maestas et al. investigated changes in the activity of the channel. Interestingly, they found a distinct divide between mutations that impact expression and those that affect functionality. Indeed, many of the mutations that altered how the channel works had no effect on surface expression. This includes a mutation that has been shown to cause Anderson Tawil Syndrome, which is characterized by developmental defects, paralysis, and heart abnormalities (Plaster et al., 2001).

Coyote-Maestas et al. then compared their results to clinical datasets containing mutations in Kir2.1 that had been observed in patients (Figure 1). Each clinically reported mutation was compared to an identical mutation obtained by the group. If the expression or function of the DMS generated mutation was higher or lower than variants which are known to be benign, it was classified as being potentially pathogenic. This allowed Coyote-Maestas et al. to predict whether variants that have not yet been clinically observed are likely to cause disease, and determine how mutations with unclear or uncertain clinical effects may impact the function and expression of Kir2.1.

The comparison also revealed that most clinically reported mutations were associated with the protein’s function rather than its ability to successfully reach and embed itself into the cell surface. As Kir2.1 is needed for many aspects of physiology, mutations that significantly impact its expression are likely to be fatal (Zaritsky et al., 2001), which may be why these types of variants are less common in the general public.

Taken together, these findings suggest that DMS can provide various mutational insights. First, it can be used to predict the clinical significance of variants with previously unknown impacts. Second, it can be used to predict regions in DNA where mutations are more likely to occur and be clinically observed. Finally, this approach can predict how a mutation will impact a protein’s cellular role. However, while this study provides a significant breadth of new information, it is worth noting that only one experiment was performed to characterize the functional consequences of the mutations studied. Additional assays are needed, including in vivo studies, to validate the pathological effect of these genetic variants.

This work demonstrates how DMS can be used to study multiple parameters. Future DMS studies will benefit from mirroring this approach, and considering how mutations impact both expression and function simultaneously. The methodology established by Coyote-Maestas et al. can be broadly applied to other ion channels, and lays the groundworks for advancing DMS studies in many biochemical applications.

## References

[bib1] Coyote-Maestas W, Nedrud D, He Y, Schmidt D (2022). Determinants of trafficking, conduction, and disease within a K^+^ channel revealed through multiparametric deep mutational scanning. eLife.

[bib2] Fowler DM, Araya CL, Fleishman SJ, Kellogg EH, Stephany JJ, Baker D, Fields S (2010). High-resolution mapping of protein sequence-function relationships. Nature Methods.

[bib3] Jackson M, Marks L, May GHW, Wilson JB (2018). The genetic basis of disease. Essays in Biochemistry.

[bib4] Jones EM, Lubock NB, Venkatakrishnan AJ, Wang J, Tseng AM, Paggi JM, Latorraca NR, Cancilla D, Satyadi M, Davis JE, Babu MM, Dror RO, Kosuri S (2020). Structural and functional characterization of G protein-coupled receptors with deep mutational scanning. eLife.

[bib5] Kunkel TA (2004). DNA replication fidelity. The Journal of Biological Chemistry.

[bib6] Li X, Ortega B, Kim B, Welling PA (2016). A common signal patch drives AP-1 protein-dependent Golgi export of inwardly rectifying potassium channels. The Journal of Biological Chemistry.

[bib7] Ma D, Taneja TK, Hagen BM, Kim BY, Ortega B, Lederer WJ, Welling PA (2011). Golgi export of the Kir2.1 channel is driven by a trafficking signal located within its tertiary structure. Cell.

[bib8] Majithia AR, Tsuda B, Agostini M, Gnanapradeepan K, Rice R, Peloso G, Patel KA, Zhang X, Broekema MF, Patterson N, Duby M, Sharpe T, Kalkhoven E, Rosen ED, Barroso I, Ellard S, Kathiresan S, O’Rahilly S, Chatterjee K, Florez JC, Mikkelsen T, Savage DB, Altshuler D, UK Monogenic Diabetes Consortium, Myocardial Infarction Genetics Consortium, UK Congenital Lipodystrophy Consortium (2016). Prospective functional classification of all possible missense variants in PPARG. Nature Genetics.

[bib9] Plaster NM, Tawil R, Tristani-Firouzi M, Canún S, Bendahhou S, Tsunoda A, Donaldson MR, Iannaccone ST, Brunt E, Barohn R, Clark J, Deymeer F, George AL, Fish FA, Hahn A, Nitu A, Ozdemir C, Serdaroglu P, Subramony SH, Wolfe G, Fu YH, Ptácek LJ (2001). Mutations in Kir2.1 cause the developmental and episodic electrical phenotypes of Andersen’s syndrome. Cell.

[bib10] Starr TN, Greaney AJ, Hilton SK, Ellis D, Crawford KHD, Dingens AS, Navarro MJ, Bowen JE, Tortorici MA, Walls AC, King NP, Veesler D, Bloom JD (2020). Deep mutational scanning of SARS-CoV-2 receptor binding domain reveals constraints on folding and ACE2 binding. Cell.

[bib11] Zangerl-Plessl EM, Qile M, Bloothooft M, Stary-Weinzinger A, van der Heyden MAG (2019). Disease associated mutations in K_IR_ proteins linked to aberrant inward rectifier channel trafficking. Biomolecules.

[bib12] Zaritsky JJ, Redell JB, Tempel BL, Schwarz TL (2001). The consequences of disrupting cardiac inwardly rectifying K(+) current (I(K1)) as revealed by the targeted deletion of the murine *Kir2.1* and *Kir2.2* genes. The Journal of Physiology.

